# Local Chemotherapy as an Adjuvant Treatment in Unresectable Squamous Cell Carcinoma: What Do We Know So Far?

**DOI:** 10.3390/curroncol28040213

**Published:** 2021-06-23

**Authors:** Luigi Bennardo, Francesco Bennardo, Amerigo Giudice, Maria Passante, Stefano Dastoli, Pietro Morrone, Eugenio Provenzano, Cataldo Patruno, Steven Paul Nisticò

**Affiliations:** 1 Unit of Dermatology, Mariano Santo Hospital, 87100 Cosenza, Italy; p.morrone@aocs.it (P.M.); e.provenzano@aocs.it (E.P.); 2 Department of Health Sciences, Magna Graecia University, 88100 Catanzaro, Italy; francesco.bennardo@studenti.unicz.it (F.B.); a.giudice@unicz.it (A.G.); maria.passante@studenti.unicz.it (M.P.); s.dastoli@materdominiaou.com (S.D.); cataldo.patruno@unicz.it (C.P.); nistico@unicz.it (S.P.N.)

**Keywords:** squamous cell carcinoma, imiquimod, 5-fluorouracil

## Abstract

*Background*: Squamous cell carcinoma (SCC) is one of the most common cancers involving skin and oral mucosa. Although this condition’s gold-standard treatment is the surgical removal of the lesions, the physician must propose alternative treatments in some cases due to the patient’s ineligibility for surgery. Among the available alternative therapies, local chemotherapy may represent an initial treatment in combination with radiotherapy or systemic chemotherapy due to the low frequency of side-effects and the lack of necessity for expensive devices. *Methods*: In this paper, we review all available literature in various databases (PubMed, Scopus-Embase, Web of Science), proposing local chemotherapy as a treatment for cutaneous and oral SCC. Exclusion criteria included ocular lesions (where topical treatments are common), non-English language, and non-human studies. *Results*: We included 14 studies in this review. The majority were case reports and case series describing the treatment of non-resectable localized SCC with either imiquimod or 5-fluorouracil. We also analyzed small studies proposing combination treatments. Almost all studies reported an excellent clinical outcome, with a low risk of relapses in time. *Conclusions*: Resection of the lesion remains the gold-standard treatment for SCC. When this approach is not feasible, local chemotherapy may represent a treatment alternative, and it may also be associated with radiotherapy or systemic chemotherapy.

## 1. Introduction

Squamous cell carcinoma (SCC) is one of the most common malignant tumors affecting the skin. The abnormal and quick growth of keratinocytes in the epidermis, often secondary to ultraviolet or sunlight exposure, is a characteristic of this cancer [1,2,3]. SCC involving the head and neck area may be particularly aggressive. Oral SCC accounts for 90% of all oral malignancies [4]. Due to its ability to metastasize, the gold-standard treatment for SCC is surgery. Some authors suggest performing surgical enlargement of the margins and radiological investigations based on the histopathologic findings [5,6,7]. However, surgical removal is not always possible for various reasons (patient ineligible for surgery, refusal of the procedure, particular areas involved, etc.) [8,9]. Clinicians usually offer an alternative treatment in these cases, such as radiation therapy or systemic chemotherapy [10,11]. Physicians may propose topical chemotherapy as an adjuvant therapy combined with other medications or, in localized in situ tumors surrounded by precancerous areas, as a primary treatment. Various topical drugs have been proposed to manage SCC, the same used for topical treatment of basal cell carcinoma (BCC) and precancerous actinic keratosis (AK) [12]. In this paper, we review all proposed topical treatments for SCC and highlight the possible therapeutic combinations available.

## 2. Materials and Methods

The authors followed criteria established in the Preferred Reporting Items for Systematic Reviews and Meta-Analyses (PRISMA) guidelines for this review [13]. 

### 2.1. PICO Question

Is topical therapy effective, in selected cases, as an alternative treatment for unresectable squamous cell carcinoma?

### 2.2. Search Strategy

Two independent researchers (L.B. and F.B.) performed a systematic review in three different databases (PubMed/Medline, Google Scholar, Scopus/Embase). Articles published up to 7 March 2021, were included. The keywords used were “topical” AND “skin cancer”, “squamous cell carcinoma”, “squamous carcinoma”, “spinous cell carcinoma”. All articles’ titles and abstracts were screened. 

### 2.3. Inclusion and Exclusion Criteria

**Figure 1:**
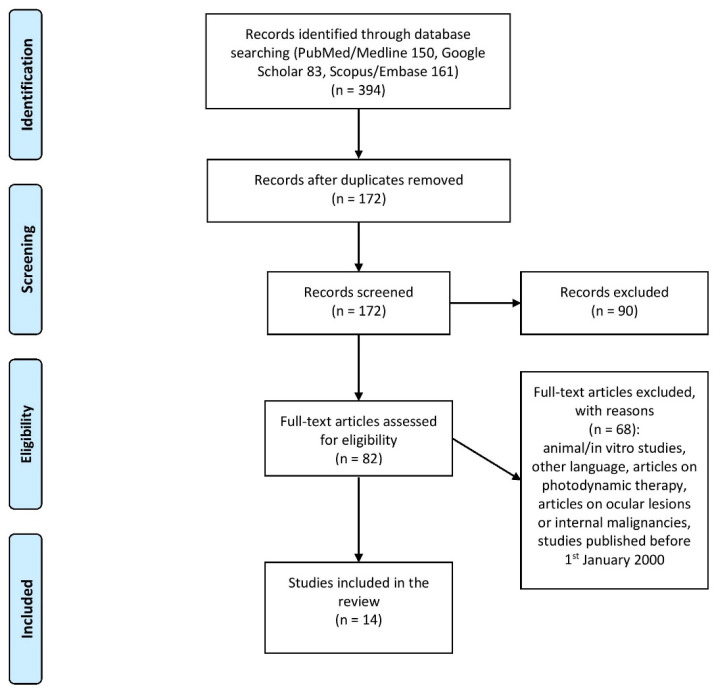
PRISMA flow chart for study selection.

Articles involving ocular lesions (in these kinds of lesions, the surgical approach is usually postponed), reporting the use of photodynamic therapy or the presence of internal malignancies, and non-human clinical studies were excluded. Articles published before January 2000 or published in languages different than English were also included. In case of discrepancies between the researchers, a third physician (E.P.) had the final word on inclusion or exclusion of the paper (Figure 1).

### 2.4. Statistical Analysis

Means, percentages, and Fisher’s exact test used within the text to assess statistical significance were all performed using SPSS Statistics version 27.0 (IBM, Armonk, New York, NY, USA).

## 3. Results

The results of the literature search are reported in the PRISMA flow diagram (Figure 1). A total of 14 papers were selected: eight case reports, four case series, and two prospective studies. Forty-nine patients were included in the review (mean age: 70.5 years old). Researchers treated 12 patients with imiquimod 5% (11 cases) or 3.75% (one case), 15 patients with 0.1 % tazarotene, and 25 patients with 5% 5-fluorouracil (four subjects were simultaneously treated with imiquimod and 5-fluorouracil). Researchers treated a patient with 80% trichloroacetic acid following intralesional administration of 5-fluorouracil. Investigators reported a complete response rate with SCC clinical and histological disappearance in 47% of lesions treated with 0.1% tazarotene, in 92% of patients treated with imiquimod, and in 96% of patients treated with 5-fluorouracil. However, given the high number of case series and case reports included in this review, the actual response rate of imiquimod and 5-fluorouracil may be much lower. The follow-up period was very variable among reported studies, ranging from 4 years to no follow-up at all. However, out of 49 patients, only a single relapse was reported 9 months after combination treatment with fractional ablative laser and 5-fluorouracil. Researchers used combination treatments in 22 out of 49 patients, managing SCC with fractional lasers and 5% 5-fluorouracil (17/22), with 5% 5-fluorouracil in the morning and 5% imiquimod at night (4/22), and with intralesional 5% 5-fluorouracil and 80% topical trichloroacetic acid (1/22). Topical combination therapies showed a response in 95% of cases, while monotherapies reported a 67% response rate, suggesting that sequential topical treatments may be more effective than single therapies alone (*p* = 0.02). The results derived from the data analysis of the studies are summarized in Table 1.

## 4. Discussion

Due to the low risk of systemic side-effects and the selective application in the area involved, topical therapies in dermatology are the most used in managing diseases [14]. Malignant tumors, however, due to their invasiveness and the risk of metastasis, are treated with surgery, and alternative therapies are only considered if the patient is not eligible or refuses the operation [15]. Our analysis of the medical literature reports that, to manage SCC, topical treatments are never used sequentially after surgery but as an alternative alone or in combination with other less invasive techniques, such as fractional lasers or intralesional injections. In these rare cases, the drugs used are mainly imiquimod and 5-fluorouracil.

Imiquimod is an immune response modifier that activates Toll-like receptor 7 and stimulates cytokines such as interferon-α, interleukin-6, and tumor necrosis factor-α. To generate an inflammatory response, this drug may activate different cells, such as natural killer cells, macrophages, B-lymphocytes, and Langerhans cells [16,17].

Imiquimod is used to treat various conditions, such as actinic keratosis, BCC, viral warts, condyloma acuminata, and molluscum contagiosum [18,19]. Various papers report the use of imiquimod in treating SCC. The main benefit of this drug seems to be a good response rate, while it may be associated with a higher risk of irritation and it may be more expensive than the other drugs.

Imiquimod has been proposed for the treatment of oral SCC with good results [20]. Furthermore, various case reports reported the use of imiquimod in immunocompromised and very old patients with optimal results [21,22,23]. This drug was proposed at 5% and 3.75% concentration [24]. Hearing loss was reported in one of the included studies as a side-effect [25].

Ondo et al. reported a case series of cutaneous SCC involving the digital areas. All patients failed topical monotherapy with imiquimod 5% and 5-fluorouracil 5%. The combination of both treatments (5-fluorouracil in the morning and imiquimod in the evening) led in 6–8 weeks to a robust inflammatory response, with the complete resolution of the clinical condition in all patients [26].

5-Fluorouracil is a chemotherapeutic agent that acts by blocking the thymidylate synthase and subsequently DNA production. The drug is parenterally administered to manage various neoplasms, such as colon, esophageal, stomach, pancreatic, breast, and cervical cancer. Its topical formulation is used for the management of precancerosis, BCC, and warts. Different authors have reported experiences in the treatment of SCC [27,28]. The main benefits of this drug are the high response rate and the fact that it is relatively cheaper than the other topical alternatives. This medication, however, is not commercially available in all countries; thus, galenic formulations in local drug stores/laboratories may not always guarantee high quality.

This drug has been proposed to treat in situ SCC of the eyelid with good results and no relapse during a 3 year follow-up [29].

Various studies proposed a combination of fractional carbon dioxide laser and 5% 5-fluorouracil under occlusion to enhance drug penetration, with a substantial reduction of the tumor mass [30]. Nguyen et al. proposed the treatment of superficial BCC and SCC with combination therapy of ablative fractional carbon dioxide laser and the subsequent topical application of 5% 5-fluorouracil under occlusion for 7 days, exploiting the cutaneous pores generated by the laser to enhance drug penetration in the skin [31]. The CO_2_ laser, due to its 10,600 nm wavelength, acts by selectively heating water and causing thermal destruction of the tissue [32,33]. The fractional CO_2_ laser can create thermal microchannels that have the goal of conveying the drug to the dermis and guaranteeing a better penetration in the area [34]. All resulting scars were histologically examined, showing a complete clearance of SCC (16 out of 16 lesions treated) and a good clearance of BCCs (10 out of 14) [35].

A subsequent follow-up study started to assess the risk of relapses after laser-assisted drug delivery of 5% 5-fluorouracil. Only 20 out of the 28 original participants of the study were recruited, and, in the 12 out of 14 SCC patients followed, only one lesion relapsed after 9 months of follow-up [36].

Viros et al. reported two cases of vemurafenib-induced multiple SCCs topically treated with 5% 5-fluorouracil with no recurrence after 11 and 18 months of follow-up [37]. Another group also reported a 67 year old woman that developed three SCCs after starting treatment with vemurafenib for cutaneous melanoma. Seven weeks of topical treatment with 5% 5-fluorouracil led to the disappearance of the lesions [38].

Vazquez et al. reported a 96 year old patient affected by a localized SCC treated with intralesional injection of 1.5 mL of 5% 5-fluorouracil and then a single layer application of 80% trichloroacetic acid. After 3 weeks, another single application of 80% trichloroacetic acid was sufficient to guarantee the clinical resolution of the lesion [39].

Various groups reported the occurrence of SCC after the application of topicals. A group reported the appearance of SCC in the mouth after the use of tacrolimus to treat oral lichen planus, although it is not clear if the insurgence of SCC was subsequent to lichen evolution or to drug application [40].

Tazarotene is a third-generation topical retinoid, a compound derived from vitamin A, used mainly to treat acne and psoriasis [41,42]. Although the drug seems to be reasonably effective, the high risk of irritation, together with the low number of experiences reported in medical literature, may preclude its use. An Italian group reported the use of daily applications of topical tazarotene for 6 months in 15 patients affected by cutaneous SSC (nine men and six women, mean age 73 years old) affecting various areas of the body (seven cases of the extremities, six of the trunk, and two of the head). Five of them were excluded during the study for various reasons, three obtained a partial remission, and only seven showed a complete histologically confirmed response [43].

All studies included in this review are summarized in Table 2.

Other topical drugs have been proposed for SCC treatment, although no in vivo study has been performed to date.

A Japanese group studied the effectiveness of a topical ointment with microRNA (miRNA) 634 combined with a systemic EGFR inhibitor in vitro and then in vivo on mice. If the findings of this study are confirmed in human studies, this combination therapy may become an alternative for cutaneous SCC ineligible for surgery [44]. 

Smith et al. treated 10 patients affected by SCC bigger than 3 cm in diameter with imiquimod three times per week for 2 weeks before surgery. A small biopsy specimen was taken from the area before starting the topical treatment. Both the biopsy and the surgical piece were histologically and immunohistochemically stained, showing an increase in the inflammatory infiltrate, with a decrease in macrophages and a T helper 1 response after topical drug therapy [45].

Different neoadjuvant treatments have been proposed to manage SCC. To our knowledge, none of these drugs has been used as a topical treatment, but all are administered in systemic form, and they include cemiplimab, pembrolizumab, cisplatin, bleomycin, and 5-fluorouracil [46].

Lastly, 5% imiquimod has been proposed as an adjuvant treatment after surgery in a SCC of the lip, with no relapse of the condition after 2 years. A prospective trial with a high number of patients will be necessary to confirm the effectiveness of this approach [47].

## 5. Conclusions

The gold-standard treatment for the management of skin and oral mucosal SCCs is complete surgical resection and, when surgery is not possible, chemotherapy and radiotherapy must be considered. In selected cases where neither of these options is available, topical drugs may represent a valid alternative due to patients’ comorbidities or refusal of aggressive treatments. If SCCs do not show aggressive behavior, if they are localized and small-sized, and if metastasis or nodal involvement is not present, this type of treatment may be considered. Among the various drugs proposed in the medical literature, 5% 5-fluorouracil seems to have a better clearance rate than imiquimod and tazarotene. However, most data were derived from case studies; hence, drug effectiveness may be lower than reported, as negative experimental results are more rarely published in literature. Further and more extensive clinical trials will be necessary to confirm the results of this review and prove the effectiveness of topicals in managing cutaneous and oral SCCs, as the low number of prospective trials present in literature does not allow drawing any final conclusions

## Figures and Tables

**Table 1:** Data analysis from the studies.

Single or Combination Therapy	Drug Used	Clearance	Recurrence
Single therapy	Imiquimod 5%	6 out of 7	No recurrence
Single therapy	Imiquimod 3.75%	1 out of 1	No recurrence
Combination therapy	5% 5-fluorouracil in the morning and 5% imiquimod at night	4 out of 4	No recurrence
Combination therapy	CO_2_ fractional laser and 5-fluorouracil	16 out of 17	1 recurrence
Combination therapy	Intralesional 5-fluorouracil, and topical trichloroacetic acid	1 out of 1	No recurrence
Single therapy	5% 5-fluorouracil	4 out of 4	No recurrence
Single therapy	0.1% tazarotene	7 out of 15	No recurrence
Total		39 out of 49	1 out of 49

**Table 2:** Studies included in the review.

Name of the Study	Type of the Study	Patients Characteristics	Topical Drug Used	Outcome
Wester et al., 2017 [20]	Case report	87 year old female, oral SCC refractory to surgery and radiotherapy	Imiquimod 5% once a day for 2 weeks, and then once a week	No recurrence of the tumor after 4 years of continuous treatment
Hengge et al., 2004 [21]	Case report	65 year old male SCC of the hair rim histologically confirmed with a biopsy	Imiquimod 5% 3 times per week overnight for 16 weeks	At week 16, biopsy of the area showed no sign of SCC
Oster-Schmidt, 2004 [22]	Case series	88year old and 92 year old women with SCC respectively of the right ear and right upper leg in poor systemic conditions.	Imiquimod 5% 5 times per week for 2 weeks	The SCCs both disappeared; no clinical reoccurrence was present
Palungwachira et al., 2005 [23]	Case series	68 year old female with high levels of arsenic showed multiple lesions on the trunk and 65 year old male in hemodialysis with a right ear helix SCC	Imiquimod 5% 3 times per week for 16 weeks	18 months after the end of treatment, no relapses were observed
Rodrigues et al., 2016 [25]	Case report	78 year old female with an SCC of the right lower cheek	Imiquimod 5% 3 times per week	Treatment was discontinued at week 3 due to the appearance of hearing loss; the condition resolved after a couple of weeks of treatment discontinuation
Bardazzi et al., 2005 [43]	Prospective study	15 patients (mean age 73 year old)	Topical tazarotene 0.1% daily for 6 months	7 out of 15 patients had a complete resolution of the condition; 3 obtained a partial response
Sharkawi et al., 2011 [29]	Case report	65 year old patient with an in situ SCC of the eyelid	5-fluorouracil 5% cream twice a day for 6 weeks	Complete disappearance of the lesion; no recurrence after 3 years follow-up
Dirschka et al., 2016 [24]	Case report	72 year old patient with a highly differentiated SCC of the vertex refusing surgery	3.75% imiquimod once daily for two 2 week treatment cycles separated by a 2 week treatment-free interval	Tumor disappearance was histologically documented; no signs of relapse at 8 month follow-up
Ondo et al., 2006 [26]	Case series	Four patients (mean age 62.5 year old) all affected by digital SCC resistant to topical monotherapy	5% 5-fluorouracil in the morning, 5% imiquimod in the evening for 8 weeks or up to strong inflammatory reaction	Complete clinical resolution of all cases
Nguyen et al., 2015 [35]	Prospective study	28 patients (mean age 71 year old) presenting 30 lesions (16 SCC, 14 BCC)	CO_2_ fractional laser followed by topical application of 5% 5-fluorouracil under occlusion for 7 days	Complete clearance of SCC (16/16) and partial clearance of BCC (10/14); one SCC relapsed after 9 months follow-up
Viros et al., 2013 [37]	Case series	Two patients affected by vemurafenib induced multiple SCCs	5% 5-fluorouracil twice a day	Resolution of the lesions and no clinical reoccurrence after 11 and 18 months
Glenn et al., 2015 [30]	Case report	53 year old patient with thick diffuse SCC of the arm resistant to topical therapies.	CO_2_ fractional laser followed by topical application of 5% 5-fluorouracil under occlusion for 7 days, then another six following occlusions without lasers	Reduction in tumor extension
Sinha et al., 2014 [38]	Case report	67 year old woman developing three SCC after starting treatment with vemurafenib	5% 5-fluorouracil once a day for 7 weeks	Clinical resolution of lesions
Vazquez et al., 2019 [39]	Case report	96 year old man with an SCC of the leg	Intralesional 1.5 mL 5 fluorouracil, and topical 80% trichloroacetic acid two times 3 weeks apart	Clinical resolution of the lesion

## Data Availability

The study did not report any new data.
